# Supplementary material for: Individual, community, and structural factors associated with linkage to HIV care among people diagnosed with HIV in Tennessee
Source: PLoS One. 2022 Mar 3;17(3):e0264508. doi: 10.1371/journal.pone.0264508 (PMC8893655; doi:10.1371/journal.pone.0264508)
Supplement: Attachment — Submitted filename: Response to Reviewers 1.26.22.doc [file pone.0264508.s002.doc]

January 26, 2022

To: PLoS ONE

We are pleased to submit our revised manuscript entitled *“Individual, Community, and Structural Factors Associated with Linkage to HIV Care Among People Diagnosed with HIV in Tennessee.”* We appreciate the comments made by the reviewers and have detailed our response to each comment below. In our response, reviewer comments are in non-italicized text, and our responses are in italicized text. Revised portions of the manuscript with the corresponding lines are included in bold text. The revised manuscript is attached with all revisions highlighted in bold text.

All authors have contributed significantly to the work and have seen and approved of this manuscript. Thank you for your consideration of our revised paper.

We thank you for your kind consideration.

Aimalohi A. Ahonkhai, MD, MPH on behalf of the co-authors

**Reviewer #1: Minor clarifications/comments:**

Comment 1. Thank you to the authors for this revised manuscript. I agree with the updated definition of linkage to care, though the text describing the linkage definition could use slight clarification. Can the authors please add more specificity to what is meant by “the first CD4 or HIV-1 RNA test result… after diagnosis”? (line 77-78). I presume you included any CD4/viral load that was collected at least 1 day after the diagnosis – if this is correct, please revise to state “after the date of diagnosis”.

*We have updated this text as suggested which now reads as below:*

The outcome of interest was linkage to HIV care, which was defined as receipt of the first CD4 or HIV-1 RNA test result captured via TN’s enhanced HIV/AIDS reporting system (eHARS) **after the date of** diagnosis, and was assessed at 30, 60, and 90 days.

Comment 2. I disagree with the statement in the discussion that “increases in teen birth rate were associated with a strong, but statistically significant increase in linkage to HIV care.” I suggest changing strong to “small”, given that the aRR is 1.02 for the association between this teen pregnancy and linkage to care.
*We agree with this sentiment and have made the suggested change. The updated text reads as below:*

Interestingly, increases in teen birth rate were associated with a **small**, statistically significant increase in linkage to HIV care.

Comment 3: Please correct the following omissions/typographical errors and re-run spell check throughout.
a. aRR for poor mental health days in the abstract is not updated with the new analysis – please check this
b. Line 212: I believe emergency room/department should be grouped with inpatient facility (as it is in Table 2). I believe “outpatient facility or emergency room” is an error here – please revise.
c. Line 253: remove inserted text “facilities ranged from 43% to”
d. Line 277: remove comma after “In addition”
e. Line 328: ‘geographic’ is misspelled

*Each of these omissions/typographical errors was addressed. Additional line editing for spelling/grammar was also completed.*

**Reviewer #2: Revised TN Linkage Study**

Comment 1. Minor. How do you interpret the teen birth rate finding? Is this a positive association: increasing teen birth rate is associated with increasing linkage to care? If so, do you think this is the result of confounding? I see this is clarified in the discussion narrative. But may want a sentence in results.
*We appreciate this comment. We have not included any interpretation of the findings in the results section, but have included potential confounding to this discussion as an explanation for this finding. The updated text is below:*

The reasons for this are not entirely clear, but could reflect intense wrap around services for pregnant and peripartum women to prevent mother-to-child HIV **transmission or could reflect potential confounding.**

Comment 2. Minor. The authors say, in the discussion and abstract, that racial disparities persisted even when adjusting for county-level social determinants of health. However, the modelling approach in the methods (and in Table 2) doesn’t appear to adjust for structural factors, just individual factors… “adjusting for a priori selected individual level covariates in multivariable analysis that were available in the surveillance data and known to be associated with the outcome of interest, including year of and age at diagnosis, sex, race/ethnicity and HIV transmission risk factor.” Does the individual model adjust for county-level social determinants? If so, please add.
*The individual model adjusts for individual-level determinants, while the county-level model adjusts for both individual and county-level determinants. We have updated the text in the Results section to more clearly present data from the county-level model that supports our assertion about racial disparities.*

**Also, in this model which adjusted for both individual and county-level factors, White and Hispanic individuals had an increased risk of 30-day linkage to care compared to Black individuals (aRR 1.33, 95%CI 1.30-1.37, aRR 1.44, 95% CI 1.41-1.47 respectively) [data not shown].**

*We have also updated Figure 2 (below) to reflect data from the county-level model (which adjusted for individual and county-level variables and included a county-by-race/ethnicity interaction term).*

We analyzed the marginal probabilities of linkage at 30-days in the four highest-burden metropolitan counties **by race/ethnicity** and found that Black patients persistently had the lowest probability of 30-day linkage to care as compared to both White and Hispanic individuals when adjusting for individual level factors, and **when adjusting for both individual and county-level factors when interacting individual-level race/ethnicity with county of residence** [Figure 2]. **Racial disparities were least prominent in Davidson County (the county seat of Nashville), which also had the highest marginal probability of linkage to care of the four highest-HIV-burdened counties.**

Comment 3. Minor – line 328, don’t follow the euphemism ‘paints a picture’. I think you’re trying to say that segregation is correlated with geographic factors? Was it specifically correlated with poor mental health and therefore correlated with poor linkage? The point you’re trying to make about segregation is poorly constructed. Suggest revising.

*We have updated the language to more clearly explain the likely relationship between racial segregation and county-level geographic factors. The updated text is in the Discussion and reads as below:*

Additionally, the high correlation of residential racial segregation (White, non-White) with many county-level factors, while not surprising, further **underscores** the relationship between race and a range of **geographic** factors that can impact health.

Comment 4. Minor. Multiple testing is not specified in the limitations, although the authors suggest it was included. Imagine this was an oversight.

*We have updated the discussion to include the limitations posed by multiple testing.*

**We acknowledge that we have included many covariates in our analyses, and could be subject to limitations from multiple testing.**
